# Leiomyosarcoma of the breast in a patient with a 10-year-history of cyclophosphamide exposure: a case report

**DOI:** 10.1186/1757-1626-1-301

**Published:** 2008-11-07

**Authors:** Jennifer De la Pena, Irene Wapnir

**Affiliations:** 1 300 Pasteur Dr. H3625 Stanford, CA 94305, USA

## Abstract

A 50 year old woman with a 10-year history of systemic lupus erythematosus (SLE) and intermittent low-dose cyclophosphamide therapy developed a palpable mass at the periphery of her left breast. Ultrasound guided core biopsy revealed a spindle cell neoplasm characterized on final pathology as a low grade leiomyosarcoma.

## Background

Sarcomas of the breast are rare entities and comprise only 1% of malignancies [1]. Leiomyosarcomas represent 2.5–6% of these and usually present as enlarging palpable masses [2]. The limited number of cases treated at any one institution has hampered making specific recommendations regarding surgery and adjuvant treatments for this pathological entity. As with other sarcomas the mainstay of treatment is resection with or without adjuvant radiotherapy.

## Case Presentation

**Figure 1:**
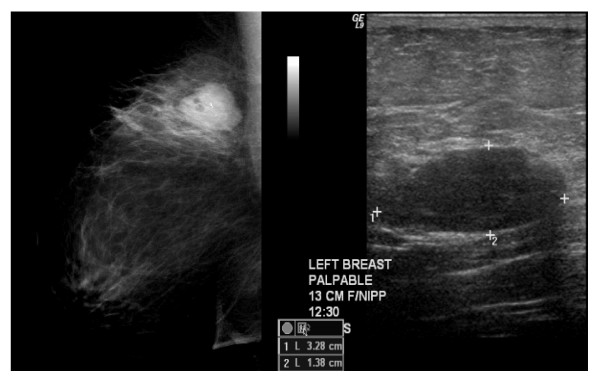
**Breast Imaging**. **(A) **Digital mammogram **(B) **ultrasound showing dominant mass in the upper central portion of the breast.

A 50 year old woman detected a mass in her left breast on self-exam (figure 1). Diagnostic mammography showed a well-circumscribed, oval mass which was hypoechoic on ultrasound and measured 3.5 × 1.4 × 2.8 cm. Family history of breast or ovarian cancer was not present. She experienced menarche at the age of 10, and early onset of menopause at age 40. She is gravida 1 and para 0, and denied past use of oral contraceptives or hormonal replacement therapy. On clinical breast exam the patient had a well circumscribed mass in her left upper outer quadrant and no regional lymphadenopathy. She then underwent ultrasound guided core biopsy which revealed a spindle cell neoplasm.

The patient's medical history was significant for SLE, diabetes mellitus type 2, rheumatoid arthritis, and asthma. Her medications include cyclophosphamide, accolade, cimetidine, etodolac, tramadol, halcion, flexeril, advair, albuterol, iron supplements, benadryl, and zofran. Her cyclophosphamide exposure was intermittent over a ten year history. The patient's past surgical history was significant for a tubal ligation and appendectomy. She currently works as an office manager and denies alcohol or tobacco use.

The diagnosis of spindle cell neoplasm raised the possibility of metaplastic carcinoma, a subtype of invasive ductal carcinoma; therefore a nodal staging procedure with sentinel lymph node biopsy was incorporated into the surgical plan. Lumpectomy was considered as there were no definite contraindications to radiotherapy with this type of SLE. However, concerns regarding cosmetic outcome and radiation tolerance in collagen vascular disease presenting with skin involvement, swayed the patient toward mastectomy. A tissue expander was placed at time of mastectomy. Unfortunately the postoperative course was complicated by wound infection and loss of expander.

**Figure 2:**
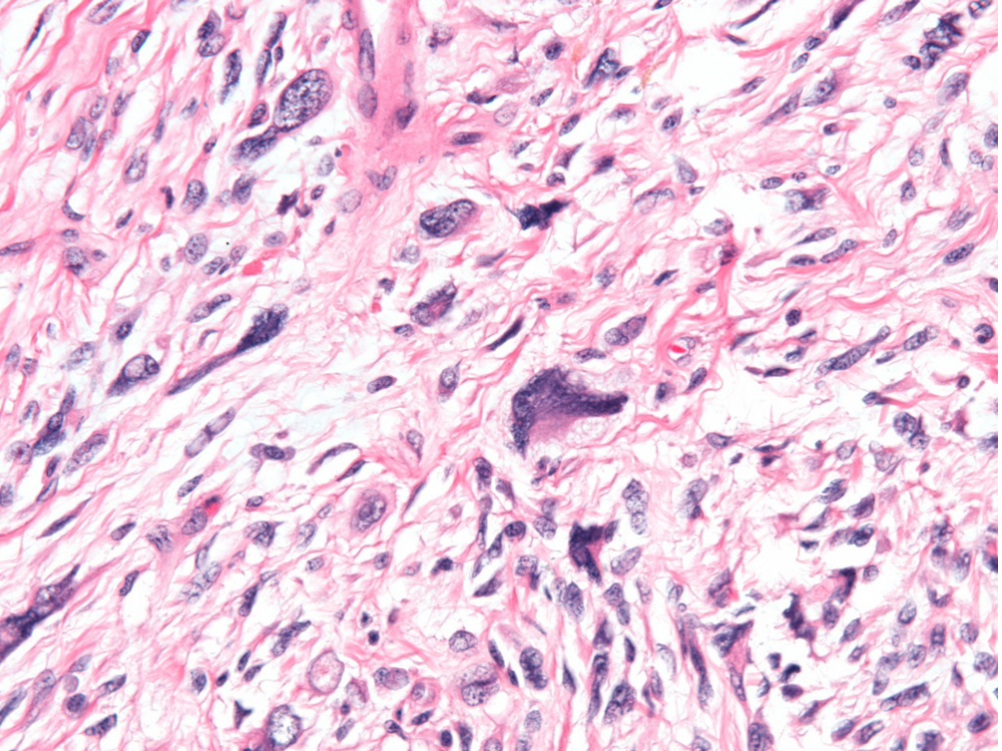
**Microphotograph of leiomyosarcoma**. Hematoxylin and eosin stain demonstrates a highly cellular, pleomorphic, and spindle shaped tumor with few mitotic figures (40×).

Final pathology revealed a 3.2 cm tumor comprised of spindle cells with abundant eosinophilic cytoplasm arranged in fascicles, with marked pleomorphism with no epithelial glandular elements (Figure 2). The tumor cells stained positive for desmin, smooth muscle actin, and calponin although negative for S100, HMB45, CKMIX, and p63: defining this lesion as a leiomyosarcoma. Metastatic workup with CT scan of the chest, abdomen, and pelvis were negative. Eleven months after her mastectomy the patient continues to do well and has no evidence of recurrent disease.

## Discussion

Leiomyosarcomas of the breast are rare neoplasms that either arise from the smooth muscle cells lining blood vessels or from stromal mesenchymal cells [2,3]. Breast sarcomas are treated by the same principles used in other sarcomas. Thus, wide local excision/lumpectomy or mastectomy with or without radiotherapy are used [4]. Lymphatic spread and nodal metastasis are not features associated with these neoplasms. Axillary nodal metastasis occurs in less than 10% of breast sarcomas, making sentinel lymph node biopsy or axillary lymph node dissection unnecessary [5]. In this case nodal staging was performed at the time of definitive surgery because the core biopsy findings (spindle cells) were interpreted as potentially representing a metaplastic breast cancer.

Cyclophosphamide is an akylating agent used commonly for the treatment of malignancies as well as for autoimmune disorders [6]. Cyclophosphamide has been linked to secondary cancers, namely bladder cancer, osteosarcoma, and leukemia [7]. Therefore it is unclear whether this type of exposure could have been a contributing factor to the development of this breast leiomyosarcoma.

Patients with collagen vascular diseases have increased late toxicity with radiotherapy, specifically increased fibrosis and thus breast irradiation is not recommended [8]. Therefore, careful consideration should be given to the potential complications of radiotherapy in patients with similar autoimmune disorders. Lastly postoperative infection is a known complication of tissue expander placement and occurs in approximately 2% of cases with associated loss [9].

## Consent

Written informed consent was obtained from the patient for publication of this case report and accompanying images. A copy of the written consent is available for review by the Editor-in-Chief of this journal.

## Competing interests

The authors declare that they have no competing interests.

## Authors' contributions

JD performed the chart review and manuscript preparation. IW evaluated, treated the patient, and participated in manuscript preparation. All authors read and approved the final manuscript.
